# Samba: Semantic segmentation of remotely sensed images with state space model

**DOI:** 10.1016/j.heliyon.2024.e38495

**Published:** 2024-09-26

**Authors:** Qinfeng Zhu, Yuanzhi Cai, Yuan Fang, Yihan Yang, Cheng Chen, Lei Fan, Anh Nguyen

**Affiliations:** a Department of Civil Engineering, Xi'an Jiaotong-Liverpool University, Suzhou, 215123, China; b Department of Computer Science, University of Liverpool, Liverpool, L69 3BX, UK; c CSIRO Mineral Resources, Kensington, WA 6151, Australia; d Department of Electrical and Electronic Engineering, Xi'an Jiaotong-Liverpool University, Suzhou, 215123, China

**Keywords:** Mamba, Semantic segmentation, Images, State space model, Remote sensing

## Abstract

High-resolution remotely sensed images pose challenges to traditional semantic segmentation networks, such as Convolutional Neural Networks (CNNs) and Vision Transformers (ViTs). CNN-based methods struggle to handle high-resolution images due to their limited receptive field, while ViT-based methods, despite having a global receptive field, face challenges when processing long sequences. Inspired by the Mamba network, which is based on a state space model (SSM) to efficiently capture global semantic information, we propose a semantic segmentation framework for high-resolution remotely sensed imagery, named Samba. Samba utilizes an encoder-decoder architecture, with multiple Samba blocks serving as the encoder to efficiently extract multi-level semantic information, and UperNet functioning as the decoder. We evaluate Samba on the LoveDA, ISPRS Vaihingen, and ISPRS Potsdam datasets using the mIoU and mF1 metrics, and compare it with top-performing CNN-based and ViT-based methods. The results demonstrate that Samba achieves unparalleled performance on commonly used remotely sensed datasets for semantic segmentation. Samba is the first to demonstrate the effectiveness of SSM in segmenting remotely sensed imagery, setting a new performance benchmark for Mamba-based techniques in this domain of semantic segmentation. The source code and baseline implementations are available at https://github.com/zhuqinfeng1999/Samba.

## 1 Introduction

**Fig. 1:**
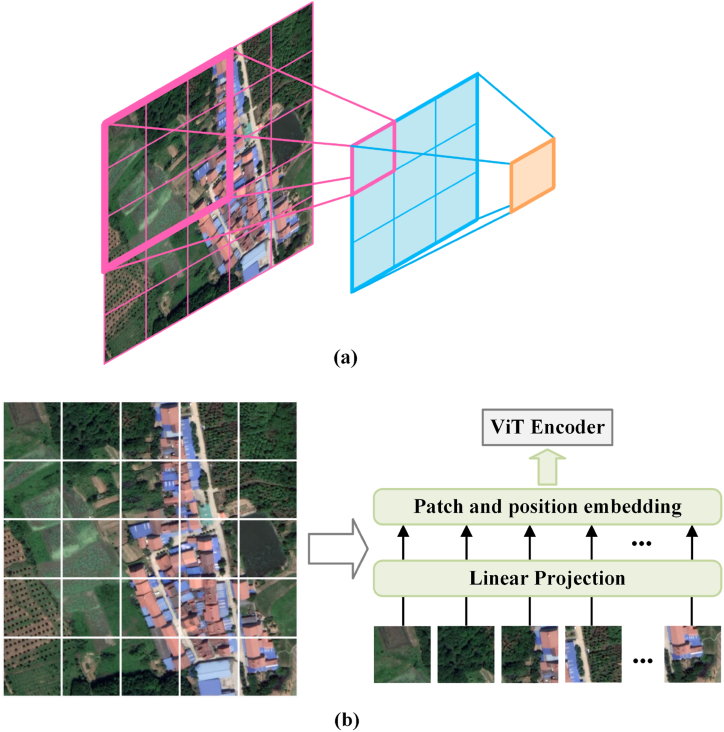
(a) CNN's receptive field, which becomes 7 × 7 after two 3 × 3 convolutions, (b) ViT slices an image into several patches, and after linear projection and embedding, multi-head self-attention calculations are performed in the encoder to possess a global receptive field.

Semantic segmentation of remotely sensed images is a crucial task in many remote sensing applications, widely implemented using deep learning methods [1]. Among these, a commonly used deep learning technique is Convolutional Neural Network (CNN) [[2], [3], [4], [5]]. By performing convolution operations that slide over image data, CNN effectively extracts semantic features from shallow to deep layers in images, serving as a cornerstone in numerous image processing tasks [[6], [7], [8]]. However, while the theoretical receptive field of CNNs can reach a global level with sufficient depth of convolutional layers, the effective receptive field remains limited in practice. This limitation poses challenges, particularly in handling high-resolution images [9], as illustrated in Fig. 1(a). Studies have shown that the effective receptive field in CNNs is significantly smaller than the theoretical receptive field, which restricts the ability to capture global context in high-resolution imagery [10,11]. Although solutions exist to mitigate this issue, they come with their own flaws. For example, while scaling images can adapt to the receptive field, it often results in resolution loss, which affects model performance. Dilated convolution can expand the receptive field, but it may lead to information loss [12,13] because of coarse feature sub-sampling. Alternatively, connecting multiple CNNs through residual connections allows for integrating high-level semantics with low-level information, thereby enhancing the model's ability to recognize different scales [14,15]. However, deep residual connections significantly increase network computation complexity.

Vision Transformer (ViT) [16] is another widely employed deep learning technique for semantic segmentation [17,18]. With its global attention mechanism, ViT overcomes the limitation posed by the receptive field and is capable of flexibly adapting to inputs of varying resolutions [19], as shown in Fig. 1(b). ViT has demonstrated remarkable performance in traditional image tasks [20,21], such as classification in ImageNet dataset [6]. However, challenges remain when employing ViT semantic segmentation of remotely sensed images. Due to the need to compute the attention mechanism between each image patch, Transformers have quadratic computational complexity. This significantly increases resource consumption when processing high-resolution images. Downsampling high-resolution remote sensing images to match the input requirements of ViT is impractical, because high resolution is often crucial for accurate perception and analysis in remote sensing tasks [22]. Furthermore, ViT requires a large amount of training data, which may be scarce in the context of remotely sensed imagery.

Recently, a novel approach called Mamba has been proposed, which utilizes a State Space Model (SSM) to capture global semantic information with low computational complexity [23]. Unlike Transformers, Mamba exhibits linear complexity, providing it with a distinct advantage in processing long sequences. Mamba is initially designed for large language models (LLMs) [24], but it's interesting to explore the effect of replacing multi-head self-attention with Mamba on vision tasks.

Inspired by Mamba, we propose Samba, a semantic segmentation framework tailored for remotely sensed images. The Samba block is specifically designed for efficient image feature extraction. In this framework, Mamba replaces multi-head self-attention in ViT to capture information from image data and is combined with multiple multi-layer perceptrons (MLP) to create a Samba block. The proposed semantic segmentation framework utilizes an encoder-decoder architecture, employing Samba blocks as the encoder and UperNet [25] as the decoder, to effectively extract multi-level semantic information.

The performance of our approach is evaluated using the LoveDA [26], ISPRS Vaihingen, and ISPRS Potsdam datasets. Comparing it against top-performing CNN and ViT methods, without loading pretrained parameters, Samba has achieved unparalleled performance on these datasets. This represents that Samba is an effective application of the State Space Model in semantic segmentation of remotely sensed images, establishing a benchmark in performance for Mamba-based techniques in this field.

The main contributions of this study can be summarized as follows.

1) We propose the Samba architecture, introducing the Mamba architecture into segmentation of remotely sensed images for the *first* time.
2) We conducted extensively comparative experiments against top-performing models, showcasing great potential of the Mamba architecture as a backbone for semantic segmentation of remotely sensed images.
3) We have established a new benchmark in performance for Mamba-based segmentation of remotely sensed images, and provided insights and potential directions for future work.

## 2 Preliminaries

### 2.1 SSM and mamba

Mamba enables SSM parameters to be functions of the input, facilitating the model to selectively propagate or discard information based on the current token. Therefore, it has attracted attention within the computer vision domain.

The core state space model of Mamba can be expressed by linear ordinary differential equations with evolution parameter A, and projection parameters B and C, as shown in Eq. (1) and (2):

(1) $$\begin{array}{c} h^{\prime }\left(t\right)=Ah\left(t\right)+Bx\left(t\right) \end{array}$$

(2) $$\begin{array}{c} y\left(t\right)=Ch\left(t\right)+Dx\left(t\right) \end{array}$$

where x(t) represents the input sequence, h(t) represents the latent state, $h^{\prime }\left(t\right)$ represents the update of the latent state, and y(t) represents the predicted output sequence. As a result, SSM can predict the state at time t based on the input sequence and the previous states.

The SSM maps x(t) to the response y(t) through the latent space of h(t). In deep learning models, the required state transition is discrete rather than continuous. Therefore, the discretization of this state is important. The discrete outputs are obtained from the sampling values based on the time step of the input results. Eq. (1) and (2) can be discretized using Eq. (3), (4), (5), (6), and (7):

(3) $$\begin{array}{c} h_{k}=\bar{A}h_{k-1}+\bar{B}x_{k} \end{array}$$

(4) $$\begin{array}{c} y_{k}=\bar{C}h_{k}+\bar{D}x_{k} \end{array}$$

(5) $$\begin{array}{c} \bar{A}=e^{\Delta A} \end{array}$$

(6) $$\begin{array}{c} \bar{B}=\left(e^{\Delta A}-I\right)A^{-1}B \end{array}$$

(7) $$\begin{array}{c} \bar{C}=C \end{array}$$

where $\bar{A}$ and $\bar{B}$ represent the discretized matrix A and B. $h_{k-1}$ represents the state of the previous timestep, and $h_{k}$ represents the state of the current timestep.

Mamba improves upon the traditional SSM by introducing a selective mechanism that allows it to remember or ignore specific inputs. Unlike SSM, which compresses all previous sequences, Mamba's approach enables the model to retain only the relevant information in long sequences.

### 2.2 mamba in vision tasks

The Mamba architecture has already demonstrated several applications in various visual tasks [27]. Vision Transformer (ViT) introduced Transformers into visual tasks by segmenting images into patches, thereby serializing them. Similarly, Vim [28] and VMamba [29] serialized images and used bidirectional and four-directional scanning followed by merging, integrating Mamba into image classification tasks and achieving results competitive with ViT. Furthermore, Mamba's application has also been witnessed in medical image segmentation. VM-unet [30] constructed a U-Net-like network using VMamba blocks, showcasing excellent performance in medical image segmentation. Similarly, U-mamba [31] incorporated Mamba blocks into a U-Net structure, further proving Mamba's effectiveness in medical image segmentation. However, the efficacy of Mamba in the semantic segmentation of remotely sensed images remains unknown, and this work aims to explore this aspect.

## 3 Methodology

### 3.1 Architecture Overview

The Samba architecture adopts an encoder-decoder structure, which is widely used in vision tasks [32] due to its simplicity and scalability. Its advantage lies in the ability to separate the feature extraction process of the encoder from the task-specific output generation of the decoder, allowing for flexible adjustments to the components and making it adaptable to different tasks and datasets [33].

**Fig. 2:**
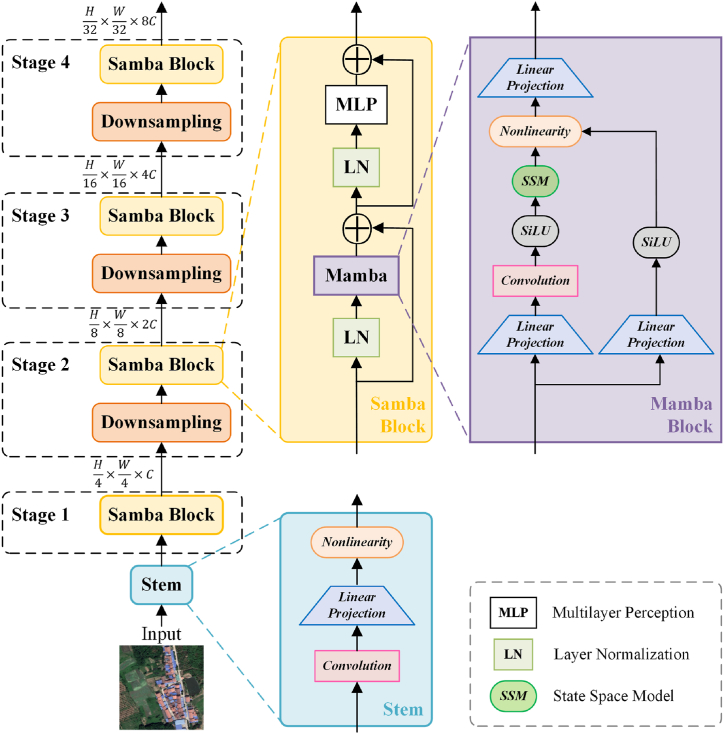
Overall architecture of Samba.

Fig. 2 illustrates the encoder architecture of Samba, featuring Samba Blocks structured into four stages for progressive downsampling. Starting with an input image of dimensions H×W×3, each Samba Block stage successively reduce its dimensions to $\frac{H}{4}\times \frac{W}{4}\times C$, $\frac{H}{8}\times \frac{W}{8}\times 2C$ , $\frac{H}{16}\times \frac{W}{16}\times 4C$, and finally $\frac{H}{32}\times \frac{W}{32}\times 8C$. These progressively reduced features are subsequently processed by the UperNet decoder, which incrementally upsamples them to produce segmentation results.

### 3.2 Samba Block

The ViT encoder uses multi-head self-attention to capture information within different representational subspaces, followed by residual connections and Layer Normalization (LN) to mitigate gradient vanishing. Subsequently, a Feed-Forward Network (FFN), consisting of an MLP and LN, is employed to introduce non-linear transformations and integrate complex information refined by multi-head self-attention.

Inspired by the robust architecture of the ViT encoder, the Samba Block adopts a similar architecture by replacing multi-head self-attention with a Mamba block. This Mamba block is utilized for feature extraction from high-resolution images, avoiding quadratic complexity in computation. In our method, a combination of the Mamba Block and MLP is adopted to enhance the model's representational capacity and strengthen its learning ability for complex data.

### 3.3 Mamba Block

Originally designed for one-dimensional sequences in natural language processing, Mamba encountered challenges when applied to the visual domain. Although some efforts [28,29] attempted to introduce Mamba into visual tasks by converting images into unfolded two-dimensional patches and scanning them in multiple directions, tests revealed that its performance still lagged behind state-of-the-art ViT methods. Zhu et al. [34] proves that when using Mamba as the backbone network, semantic segmentation of remotely sensed images does not rely on multi-directional scanning strategies. In our proposed architecture, we do not scan the image patches in this way, and we linearly project the image's flattened patches in a manner similar to ViT. Specifically, we utilize a combination of convolutional layers, projection layers, and layer normalization for the input of the Mamba Block. This block is constructed based on the H3 architecture [35], a well-known SSM architecture, combined with a Gated MLP, as shown on the right side of Fig. 2. The sequence length of flattened patches derived from high-resolution remote sensing imagery is substantial. Mamba models this extensive sequence, endowing each patch with semantic information induced from the contextual sequence. Consequently, this approach facilitates the effective extraction of features from high-resolution remote sensing images.

## 4 Experiments

### 4.1 Datasets and metrics

Our proposed framework is validated using three commonly used benchmark datasets, including LoveDA, ISPRS Vaihingen, and Potsdam datasets, as detailed as follows.

LoveDA consists of remotely sensed RGB imagery with a spatial resolution of 0.3 m, including 7 semantic classes. Its image resolution is 1024 × 1024. It consists of 2522 training images, 1669 validation images, and 1796 test images, with the validation set used for performance evaluation.

ISPRS Vaihingen consists of remotely sensed imagery with a spatial resolution of 9 cm, and includes 6 semantic classes. Its average image resolution is 2494 × 2064. Near infrared (IR), red, and green channels are used in our experiment. We use 17 images with tiles of 2, 4, 6, 8, 10, 12, 14, 16, 20, 22, 24, 27, 29, 31, 33, 35, 38 for testing (the same set of images for validation too) in this study, with the remaining 16 images allocated for training. The clutter class is not calculated in evaluation.

ISPRS Potsdam consists of remotely sensed imagery with a spatial resolution of 5 cm, featuring 6 semantic classes. Each image has a resolution is 6000 × 6000. Red, green, and blue (RGB) channels are used in our experiments. For testing and validation, we use 14 images with ID 2_13, 2_14, 3_13, 3_14, 4_13, 4_14, 4_15, 5_13, 5_14, 5_15, 6_13, 6_14, 6_15, 7_13 in this study. Training is implemented using the remaining 24 images, following the same setting as SBSS [5]. The clutter class is not calculated in evaluation.

The Mean Intersection over Union (mIoU) is used to assess the accuracy of segmentation, and is calculated using Eq. (8):

(8) $$\begin{array}{c} \text{mIoU}=\frac{1}{C}\sum_{c=1}^{C}\frac{{TP}_{c}}{{TP}_{c}+{FP}_{c}+{FN}_{c}} \end{array}$$

where ${TP}_{c}$, ${FP}_{c}$, and ${FN}_{c}$ respectively represent the true positives, false positives, and false negatives for class c, elobarated as follows:

True Positives (${TP}_{c}$): These are the pixels or areas correctly identified as belonging to class c. It means that both the predicted label and the true label agree on class c.

False Positives (${FP}_{c}$): These refer to the pixels or areas incorrectly labeled as class c by the model, but in reality, they belong to a different class. This error type reflects overestimation of class c presence.

False Negatives (${FN}_{c}$): These are the pixels or areas that truly belong to class c but were missed or incorrectly labeled as another class by the model. This represents an underestimation of class 's presence.

In addition to mIoU, we also use the Mean F1-Score (mF1) to evaluate segmentation performance. mF1 also considers ${TP}_{c}$, ${FP}_{c}$, and ${FN}_{c}$, but it focuses on the harmonic mean of precision and recall to mitigate the effects of imbalanced class distributions. mF1 is calculated using Eq. (9):

(9) $$\begin{array}{c} \text{mF}1=\frac{1}{C}\sum_{c=1}^{C}\frac{2\times {TP}_{c}}{{2\times TP}_{c}+{FP}_{c}+{FN}_{c}} \end{array}$$

### 4.2 Training settings

In this study, we evaluate our method against several established approaches known for their effectiveness. These include CNN-based methods such as ConvNeXt [36], ResNet [37], Deeplab V3+ [14], and PSPNet [38], and ViT-based methods such as Swin-T [39], and Segformer [40]. To ensure fair comparisons, the tested methods are uniformly initialized without their pre-trained parameters, and input images are cropped into 512 × 512. The optimization and learning rate strategy settings for these methods adhere to widely adopted optimal configurations. Cross entropy is used as the loss function. Data augmentation can effectively enhance the generalization ability of deep learning models when training data is limited [41], so we use random resize, random crop, random flip, and photo metric distortion to augment training data. Specific training settings are summarized in Table 1. All experiments are conducted using two NVIDIA RTX 3090 GPUs (24G) and two 4090D GPUs (24G).

**Table 1:** Training settings for semantic segmentation networks.

Decoder	Encoder	Total training iterations	Batch size	Optimizer	Initial learning rate	Warmup iterations	Learning rate schedule	Weight decay	Data augmentation
DeepLab V3+ [14]	ResNet50 [37]	15k	16	SGD	0.01	NaN	PolyLR	0.0005	Random Resize, Random Crop, Random Flip, Photo Metric Distortion
PSPNet [38]	ResNet50 [37]								
UperNet [25]	ResNet50 [37]								
UperNet [25]	ConvNeXt [36]			AdamW	0.0001	1500		0.05	
UperNet [25]	Swin-T [39]				0.000006			0.1	
Segformer [40]	Mix ViT [40]	160k			0.00006			0.01	
UperNet [25]	Samba (Ours)	15k			0.0006			0.01	

### 4.3 Results

The experimental results are summarized in Table 2, Table 3, Table 4, highlighting the performance of Samba against the current top-performing CNN-based and ViT-based methods. As each decoder-encoder experiment in our study was iterated three times, the result for each metric in Table 2, Table 3, Table 4 represents the average value obtained from the three repeated experiments. Samba achieved the best performance on the LoveDA, ISPRS Vaihingen, and ISPRS Potsdam datasets, significantly surpassing CNN-based methods and slightly exceeding the ViT-based methods. Specifically for LoveDA, when using UperNet as the decoder, Samba outperformed the best-performing ViT-based model Segformer by 3.95 % in the mIoU metric, and surpassed the best-performing CNN-based model ConvNeXt by 10.3 % in terms of the mIoU metric. Specifically, Samba demonstrated the most significant improvements in the Building, Water, and Agricultural categories, with improvements of 7.96 %, 7.73 %, and 4.96 %, respectively, compared to the most effective method among those compared.

**Table 2:** Accuracy of semantic segmentation on the LoveDA dataset from Samba and other compared methods. The accuracy of each category is presented by the IoU metric. The highest scores are highlighted in bold.

Decoder	Encoder	mIoU	mF1	BG	BU	RO	WA	BA	FO	AG
UperNet [25]	ConvNeXt [36]	36.81	52.97	45.51	30.48	43.53	49.17	17.09	35.19	36.70
UperNet [25]	ResNet50 [37]	32.86	48.34	32.23	48.15	38.97	37.66	14.17	19.26	39.58
DeepLab V3+ [14]	ResNet50 [37]	34.60	50.72	43.55	47.32	41.17	39.41	22.43	20.69	36.65
PSPNet [38]	ResNet50 [37]	33.73	49.85	36.30	48.81	35.03	36.34	21.10	23.74	34.82
UperNet [25]	Swin-T [39]	41.08	57.52	49.78	49.58	40.58	52.12	22.61	34.87	37.98
Segformer [40]	Mix ViT [40]	43.16	59.75	50.71	49.89	46.12	47.79	**23.95**	**41.03**	42.60
UperNet [25]	**Samba (Ours)**	**47.11(1st)**	**63.17**	**51.71**	**57.85**	**49.86**	**59.85**	21.93	41.00	**47.56**

BG: Background, BU: Building, RO: Road, WA: Water, Barren: BA, FO: Forest, AG: Agricultural.

**Table 3:** Accuracy of semantic segmentation on the ISPRS Vaihingen dataset from Samba and other compared methods. The accuracy of each category is presented by the IoU metric. The highest scores are highlighted in bold.

Decoder	Encoder	mIoU	mF1	Impervious surface	Building	Low vegetation	Tree	Car
UperNet [25]	ConvNeXt [36]	67.42	79.57	76.89	81.71	60.48	73.11	44.01
UperNet [25]	ResNet50 [37]	70.25	81.70	79.18	83.35	65.76	77.48	45.52
DeepLab V3+ [14]	ResNet50 [37]	69.10	80.78	77.89	82.63	64.85	76.76	43.38
PSPNet [38]	ResNet50 [37]	68.72	80.29	78.80	82.84	65.54	77.11	39.30
UperNet [25]	Swin-T [39]	71.72	82.78	80.43	85.18	**67.30**	77.69	48.01
Segformer [40]	Mix ViT [40]	70.23	81.66	79.38	85.77	64.13	75.87	46.01
UperNet [25]	**Samba (Ours)**	**73.56(1st)**	**84.23**	**81.67**	**87.26**	65.82	**77.93**	**55.10**

**Table 4:** Accuracy of semantic segmentation on the ISPRS Potsdam dataset from Samba and other compared methods. The accuracy of each category is presented by the IoU metric. The highest scores are highlighted in bold.

Decoder	Encoder	mIoU	mF1	Impervious surface	Building	Low vegetation	Tree	Car
UperNet [25]	ConvNeXt [36]	74.70	85.22	79.57	85.64	66.79	62.09	79.42
UperNet [25]	ResNet50 [37]	74.98	85.54	78.11	82.52	68.97	65.87	79.42
DeepLab V3+ [14]	ResNet50 [37]	75.23	85.70	78.37	82.76	68.32	66.54	80.18
PSPNet [38]	ResNet50 [37]	73.13	84.32	77.49	82.02	67.10	64.47	74.47
UperNet [25]	Swin-T [39]	76.46	86.46	80.97	86.45	70.20	66.03	78.67
Segformer [40]	Mix ViT [40]	81.13	89.42	83.40	89.63	72.74	73.38	86.51
UperNet [25]	**Samba (Ours)**	**82.29(1st)**	**90.15**	**84.45**	**90.06**	**74.37**	**74.98**	**87.61**

Despite significant differences in training data quantities, spatial resolutions, and segmentation categories among the considered datasets, similar results were also achieved on the ISPRS Vaihingen and ISPRS Potsdam datasets. Samba surpassed other methods in terms of the mIoU and mF1 metrics as well as the IoU for most of the segmentation categories. When combined with the UperNet decoder, Samba utlized lower FLOPs per patch and fewer parameters than Swin-T, ResNet50, and ConvNeXt, as shown in Table 5 Specifically, Samba achieved a FLOP count of 232 G and 51.9 M parameters, which was lower than Swin-T's 236 G FLOPs and 58.9 M parameters, ResNet50's 237 G FLOPs and 64.0 M parameters, and ConvNeXt's 234 G FLOPs and 59.2 M parameters.

**Table 5:** Comparison of computational complexity and parameters between Samba and other compared methods.

Decoder	Year	Encoder	Year	Flops per patch (G)	Parameters (M)
UperNet [25]	2018	ConvNeXt [36]	2022	234	59.2
UperNet [25]	2018	ResNet50 [37]	2016	237	64.0
DeepLab V3+ [14]	2018	ResNet50 [37]	2016	177	41.2
PSPNet [38]	2017	ResNet50 [37]	2016	179	46.6
UperNet [25]	2018	Swin-T [39]	2021	236	58.9
Segformer [40]	2021	Mix ViT [40]	2021	8	3.7
UperNet [25]	2018	**Samba (Ours)**	2024	232	51.9

While SegFormer is a network focused on lightweight design with significantly lower FLOPs per patch (8 G) and parameters (3.7 M) as shown in Table 5 and it also requires more training iterations to achieve comparable performance. This training requirement makes its parameters and computational load less suitable for direct comparison with standard, full-scale networks. Samba's FLOPs and parameter count indicate that it has a competitive computational burden and segmentation performance compared to other state-of-the-art models, making it well-suited for high-resolution image analysis applications. It is worth noting that the current Samba architecture still has room for optimization, and with further engineering improvements, there is potential to reduce its computational burden. The SSM design also demonstrates its potential in lightweight models. For example, Pei et al. [42] have made preliminary attempts at lightweighting SSM and achieved competitive results in image classification tasks.

**Fig. 3:**
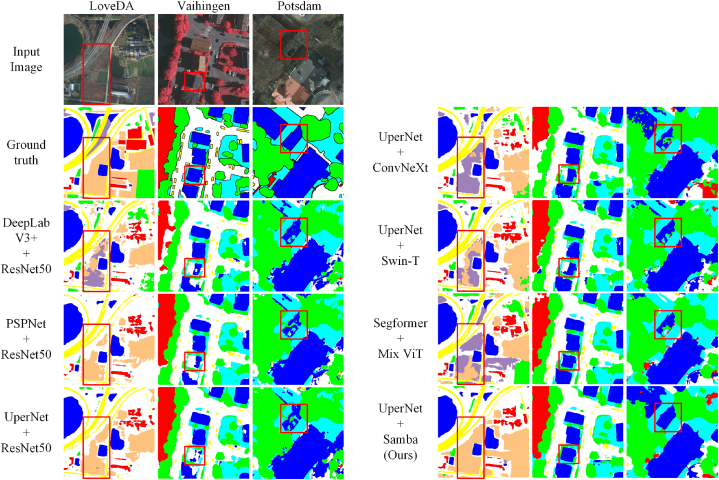
Visual comparisons of segmentation outcomes between Samba and other compared methods.

Moreover, as shown in Fig. 3, we visualized the semantic segmentation results achieved by Samba along with those compared models on the LoveDA, ISPRS Vaihingen, and ISPRS Potsdam dataset. These visualizations further demonstrate Samba's consistently enhanced performance in semantically segmenting high-resolution remotely sensed images.

## 5 Discussion

The findings of this study highlight the effectiveness of Mamba in semantically segmenting high-resolution remotely sensed images, pushing forward state-of-the-art accuracy while demanding lower computational resources compared to other methods considered. Although the performance of Mamba approaches that of ViT in visual tasks, the newly proposed Samba outperforms ViT in semantic segmentation. This superior performance is attributed to the architecture of Mamba, which is exceptionally suited for this type of tasks, demonstrating its capability in efficiently capturing global semantic information.

Observations in Fig. 3 reveal that semantic segmentation results of CNN-based methods exhibit a notable dispersion across local regions, manifesting in a tendency towards fragmented segmentation. Additionally, segmentation results exhibit blurred segmentation boundaries, and incomplete segmentation of large-scale objects. This is due to the inherent limitation of CNNs with fixed receptive fields. Although adept at extracting local features, they struggle to effectively capture large-scale contextual information when dealing with high-resolution remotely sensed images, leading to a piecemeal recognition of features within the scene. Simultaneously, CNN-based methods frequently encounter false positive errors in large-scale objects due to their deficiency in understanding context. It underscores the challenge faced by CNN-based methods in achieving holistic and contiguous segmentation outcomes, particularly in complex remote sensing imagery where contextual coherence and spatial continuity are crucial for accurate land cover and land use classification. Compared to CNN-based methods, ViT-based methods exhibit smoother segmentation results but also suffer from the issue of blurred boundaries. Despite their advantage in understanding the global context, ViT sacrifices the fidelity and precision of capturing local features. The manifestation of false positives in localized areas underscores a critical challenge for ViT models in balancing the extraction and integration of global and local cues to achieve accurate and reliable segmentation when faced with high-resolution remotely sensed images.

Compared to the LoveDA dataset, which comprises 2522 training images, the training data available for the ISPRS Vaihingen and ISPRS Potsdam datasets are considerably limited. Both CNN-based and ViT-based methods exhibit a pronounced issue of FN errors in these datasets, especially in larger areas, such as the building class (indicated by blue in Fig. 3 for Vaihingen and Potsdam). Conversely, Samba manages to achieve relatively complete segmentation results even with limited training data, especially in the area within the red boxes in Fig. 3. While CNN-based and ViT-based methods can produce relatively complete segmentation outcomes on the LoveDA dataset, they manifest significant FP errors in larger regions, for instance, the agricultural areas (represented by light apricot color in Fig. 3 of LoveDA) are categorized into barren areas (represented by the light purple color), whereas Samba seldom encounters such errors in the segmentation of large areas. The Agricultural category was both crucial and challenging to identify within the LoveDA dataset. Samba's exemplary performance in this category underscores the effectiveness of its architecture in extracting information through context induction in the semantic segmentation of high-resolution remote sensing imagery.

**Fig. 4:**
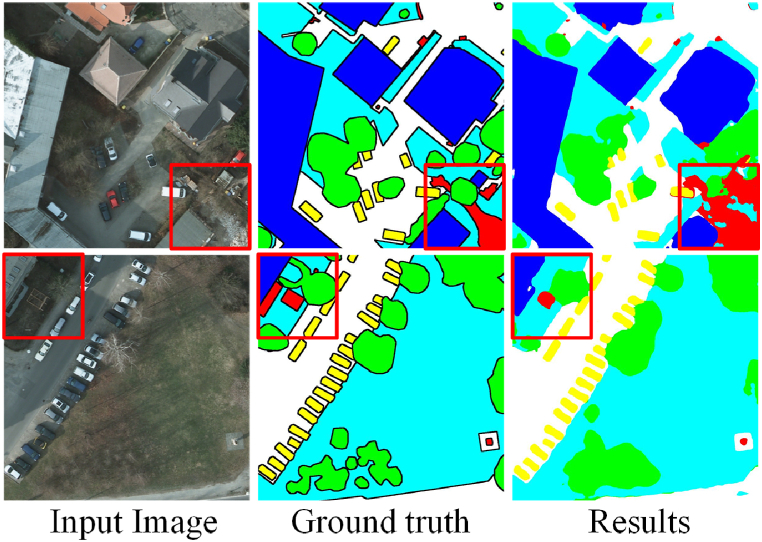
Visual results of Samba's limitation in local regions.

Thanks to the SSM's powerful inductive capability in long sequences, Samba exhibited outstanding segmentation results in the dataset considered. Samba delivered more complete and accurate segmentation of large terrain areas, compared to other methods, thus performing well in minimizing false positives. Due to the architectural similarity between Samba and ViT, the characteristics of Samba's segmentation results are deemed similar to those of ViT. Like ViT, Samba's limitations are reflected in its insufficient attention to detail in certain regions. For example, Fig. 4 illustrates Samba's limitations in handling detailed areas. When the local features are complex or involve multiple classes, as seen in the red-boxed regions in Fig. 4, Samba tends to produce false positives and false negatives, with less accurately segmented boundaries of objects. This is because the Samba architecture focuses more on modeling the global information relationships between image patches, which leads to information loss in the aggregation of local features, resulting in less precise segmentation outcomes in these local areas. This observation points to the need for additional strategies to mitigate the impact of overgeneralized feature attribution on segmentation performance.

Based on our research, we suggest the following directions for future investigations.

1) Despite its strengths in long-sequence modeling and low computational complexity, Samba, like ViT, has limited capability in extracting local information. This may result in Samba being less effective in handling semantic segmentation tasks involving small objects. Given the advantages of CNNs in extracting details, future work could explore combining Samba with CNNs to enhance the capability of capturing fine details.
2) The challenge of object scale variation in remote sensing image data necessitates improving Samba's adaptability to scale changes in future work. Future improvements to the Samba architecture could draw inspiration from the Swin Transformer approach, utilizing shifted windows to model internal patches within local regions. This would enable flexible handling of objects at different scales, ensuring a better integration of local and global information.
3) Given the limited access to annotated remote sensing image data, transfer learning is considered an important technique for segmentation tasks. The pre-trained models can be obtained after training on large-scale datasets, such as ImageNet [6]. Exploring efficient and effective transfer learning methods tailored to the Samba architecture is also potential research direction.
4) Since Samba performs well in dealing with long sequences, it is valuable to explore their application in semantic segmentation of multi-channel data [43,44], such as hyperspectral data. Given the scalability of the encoder-decoder architecture, it would be valuable for future work to explore the potential of the Samba architecture in other remote sensing image tasks, such as small object detection and change detection.

## 6 Conclusion

This article introduces Samba, a novel semantic segmentation framework built on Mamba, specifically designed for segmentation of high-resolution remotely sensed images, marking the first integration of Mamba within the domain.

By evaluating its performance on the LoveDA, ISPRS Vaihingen, and ISPRS Potsdam datasets, Samba surpassed stat-of-the-art CNN-based and ViT-based methods on all those three datasets. Particularly for LoveDA, with UperNet serving as the decoder, Samba exceeded the performance of Segformer (a leading ViT-based model) and ConvNeXt (a top-performing CNN-based model) by 3.95 % and 10.3 %, respectively, in terms of the mIoU metric. Samba's limitation lies in its limited performance in local regions with complex details, as it focuses more on global information modeling, leading to potential information loss in local feature aggregation. These set new benchmarks in performance and demonstrating the effectiveness and potential of the Mamba architecture in semantic segmentation of high-resolution remote sensing imagery.

## Data availability statement

All data are fully available without restriction.

## Funding

This work was supported in part by the Xi'an Jiaotong-Liverpool University Research Enhancement Fund under Grant REF-21-01-003, and in part by the Xi'an Jiaotong-Liverpool University Postgraduate Research Scholarship under Grant FOS2210JJ03.

## CRediT authorship contribution statement

**Qinfeng Zhu:** Writing – review & editing, Writing – original draft, Visualization, Methodology, Investigation, Data curation, Conceptualization. **Yuanzhi Cai:** Investigation, Conceptualization. **Yuan Fang:** Visualization, Investigation. **Yihan Yang:** Validation. **Cheng Chen:** Investigation. **Lei Fan:** Writing – review & editing, Supervision, Funding acquisition. **Anh Nguyen:** Conceptualization.

## Declaration of competing interest

The authors declare that they have no known competing financial interests or personal relationships that could have appeared to influence the work reported in this paper.
